# Nongenomic modulation of the large conductance voltage‐ and Ca^2+^‐activated K^+^ channels by estrogen: A novel regulatory mechanism in human detrusor smooth muscle

**DOI:** 10.14814/phy2.13351

**Published:** 2017-07-01

**Authors:** Kiril L. Hristov, Shankar P. Parajuli, Aaron Provence, Eric S. Rovner, Georgi V. Petkov

**Affiliations:** ^1^ Department of Drug Discovery and Biomedical Sciences South Carolina College of Pharmacy University of South Carolina Columbia South Carolina; ^2^ Medical University of South Carolina Charleston South Carolina

**Keywords:** 17*β*‐estradiol, patch‐clamp, paxilline, urinary bladder smooth muscle

## Abstract

Estrogens have an important role in regulating detrusor smooth muscle (DSM) function. However, the underlying molecular and cellular mechanisms by which estrogens control human DSM excitability and contractility are not well known. Here, we used human DSM specimens from open bladder surgeries on 27 patients to elucidate the mechanism by which 17*β*‐estradiol regulates large conductance voltage‐ and Ca^2+^‐activated K^+^ (BK) channels, the most prominent K^+^ channels in human DSM. We employed single BK channel recordings on inside‐out excised membrane patches, perforated whole‐cell patch‐clamp on freshly isolated DSM cells, and isometric tension recordings on DSM‐isolated strips to investigate the mechanism by which 17*β*‐estradiol activates BK channels. 17*β*‐Estradiol (100 nmol/L) rapidly increased depolarization‐induced whole‐cell K^+^ currents in DSM cells. The 17*β*‐estradiol stimulatory effects on whole‐cell BK currents were completely abolished by the selective BK channel inhibitor paxilline (1 *μ*mol/L), clearly indicating that 17*β*‐estradiol specifically activates BK channels. 17*β*‐Estradiol also increased the frequency of ryanodine receptor‐mediated transient BK currents. Single BK channel recordings showed that 17*β*‐estradiol (100 nmol/L) significantly increased the BK channel open probability of inside‐out excised membrane patches, revealing that 17*β*‐estradiol activates BK channels directly. 17*β*‐Estradiol reduced spontaneous phasic contractions of human DSM‐isolated strips in a concentration‐dependent manner (100 nmol/L‐1 *μ*mol/L), and this effect was blocked by paxilline (1 *μ*mol/L). 17*β*‐Estradiol (100 nmol/L) also reduced nerve‐evoked contractions of human DSM‐isolated strips. Collectively, our results reveal that 17*β*‐estradiol plays a critical role in regulating human DSM function through a direct nongenomic activation of BK channels.

## Introduction

Complex and coordinated regulatory mechanisms involving hormones, neurotransmitters, receptors, and ion channels regulate the physiology and pathophysiology of detrusor smooth muscle (DSM) (Andersson and Wein 2004; Petkov 2012, 2014). Increasing evidence suggests that sex hormones, specifically an estrogen 17*β*‐estradiol, have a critical role in the control of DSM function (Lin et al. 2006; Valeri et al. 2009; Petkov 2014). Yet, the molecular and cellular mechanisms underlying 17*β*‐estradiol‐mediated regulation of human DSM physiology have not been fully elucidated. Estrogen receptors are expressed in the smooth muscle of the bladder, urethra, vagina, and pelvic floor (Robinson et al. 2013); however, controversy in the literature concerning the role of estrogens in urinary bladder function exists. For example, estrogens have been shown to stimulate DSM contractility in rabbits (Lin et al. 2006), while DSM relaxation was reported in pigs (Dambros et al. 2004), rats (Valeri et al. 2009), and guinea pigs (Yasay et al. 1995).

Current research has suggested a potential role for estrogen replacement therapies in the treatment of bladder disorders including overactive bladder (OAB) and urinary incontinence (Smith and Wein 2010; Robinson et al. 2013, 2014). Some studies have reported positive results for estrogen therapies in mitigating symptoms of OAB in postmenopausal women, while other studies reported the contrary (Robinson et al. 2013, 2014; Hanna‐Mitchell et al. 2016). Large epidemiological studies investigating the use of systemic hormone replacement therapy in the prevention of cardiovascular disease and osteoporosis revealed that these therapies may increase the risk for development of urinary incontinence (Grady et al. 2001; Grodstein et al. 2004; Hendrix et al. 2005). However, more recent meta‐analysis supported the use of local, but not systemic, estrogen therapies for the treatment of urge urinary incontinence and OAB (Nappi and Davis 2012). Therefore, an improved understanding of the roles of estrogens in bladder function may lead to greater insight concerning the efficacy of estrogen replacement therapies for OAB treatment.

Ion channels are particularly important targets for mediating the effects of estrogens. For example, 17*β*‐estradiol has been shown to interact with K^+^ channels, including voltage‐gated K^+^ and TASK channels, the latter being a member of the two‐pore domain K^+^ channel family (Druzin et al. 2011; Hao and Li 2014). Furthermore, 17*β*‐estradiol has been reported to activate the large conductance voltage‐ and Ca^2+^‐activated K^+^ (BK) channels in some smooth muscles including cultured human coronary artery smooth muscle cells (White et al. 2002) and colonic myocytes (Dick and Sanders 2001; Dick et al. 2001). As BK channels are fundamental regulators of DSM function under normal and OAB conditions, when the channel expression is altered, pharmacological modulation of their activity could potentially be an effective approach to treat forms of lower urinary tract dysfunction, such as OAB (Petkov 2012, 2014; Hanna‐Mitchell et al. 2016).

A recent study revealed that 17*β*‐estradiol activates BK channels in guinea pig DSM cells through direct nongenomic mechanisms (Provence et al. 2015). However, the functional interaction between 17*β*‐estradiol and BK channels in human DSM remains unknown. Significant species‐related differences between experimental animal and human DSM excitability and contractility have been well documented (Hashitani and Brading 2003). Moreover, studies on human DSM cells and tissues are of critical importance to validate the findings obtained from animal models.

In this study, we used a multidisciplinary experimental approach including amphotericin‐B‐perforated whole‐cell patch‐clamp electrophysiology, single BK channel recordings, and isometric DSM tension recordings to test the hypothesis that 17*β*‐estradiol decreases the excitability and contractility of human DSM via a mechanism involving direct BK channel activation.

## Materials and Methods

### Human DSM specimen collection

This study was conducted according to protocol Pro00045232, reviewed and approved by the Medical University of South Carolina Institutional Review Board. Human bladder specimens were obtained from 27 patients (20 males and seven females, average age of 69.0 ± 2.1 years, 25 Caucasians and two Hispanic), who did not have a preoperative history of OAB. The DSM tissue samples were obtained during open bladder surgeries performed for a variety of indications such as bladder cancer, including radical cystectomy for urothelial carcinoma, and adenocarcinoma. In such cases, the collected DSM tissue was remote from the site of tumor. DSM strips were dissected from the bladder specimens, and after removing the urothelial and lamina propria layers, the strips were used for single DSM cell isolation and isometric DSM tension recordings.

### Isometric DSM tension recordings

Isometric DSM tension recordings of human DSM‐isolated strips (~7–8 mm long and ~4–5 mm wide) were conducted as previously described (Hristov et al. 2011, 2012).

### DSM single‐cell isolation

Human single DSM cells were enzymatically isolated as previously described (Hristov et al. 2011, 2012; Parajuli et al. 2015). Freshly isolated human DSM cells were used in the experiments within 8–10 h following cell isolation.

### Patch‐clamp electrophysiological experiments

Patch‐clamp experiments were performed as previously described (Hristov et al. 2011, 2012; Malysz et al. 2013; Parajuli et al. 2015). We applied the amphotericin‐B‐perforated whole‐cell patch‐clamp technique to record transient BK currents (TBKCs), depolarization‐induced steady‐state whole‐cell BK currents, and the resting membrane potential (RMP) of human freshly isolated DSM cells. TBKCs in DSM cells were recorded at the holding potential of −20 mV (corrected for junction potential). Depolarization‐induced steady‐state whole‐cell BK currents were elicited by holding the DSM cells at −70 mV and then brief, 200 msec depolarization steps were applied from −40 mV to +80 mV in increments of 20 mV. Single BK channel activity was recorded from inside‐out excised membrane patches using symmetrical K^+^ (140 mmol/L) solutions, at a holding potential of −60 mV (V_h_ = −60 mV). The electrochemical driving force for K^+^ in these experimental conditions was +60 mV (V_DV_ = 60 mV). An eight‐pole Bessel filter 900CT/9L8L (Frequency Devices, Ottawa, IL) was used to filter the currents for the single channel recordings. Paxilline (1 *μ*mol/L), a selective BK channel inhibitor, was used to dissect their functional role in mediating 17*β*‐estradiol‐induced effects on DSM function. All patch‐clamp experiments were conducted at room temperature (22–23°C).

### Solutions and drugs

Ca^2+^‐free dissection solution contained (in mmol/L): 80 monosodium glutamate, 55 NaCl, 6 KCl, 10 glucose, 10 HEPES, 2 MgCl_2_, and the pH was adjusted to 7.3 with NaOH. For the functional studies on human DSM contractility, the physiological saline solution had the following composition (in mmol/L): 119 NaCl, 4.7 KCl, 24 NaHCO_3_, 1.2 KH_2_PO_4_, 2.5 CaCl_2_, 1.2 MgSO_4_, and 11 D‐glucose, aerated with 95% O_2_/5% CO_2_ (pH 7.4). Extracellular (bath) solution used for the perforated whole‐cell patch‐clamp experiments contained (in mmol/L): 134 NaCl, 6 KCl, 1 MgCl_2_, 2 CaCl_2_, 10 glucose, 10 HEPES, and pH was adjusted to 7.4 with NaOH. The patch‐pipette solution for the perforated patch‐clamp experiments contained (in mmol/L): 110 potassium aspartate, 30 KCl, 10 NaCl, 1 MgCl_2_, 10 HEPES, 0.05 EGTA, and pH was adjusted to 7.2 with NaOH. Amphotericin‐B stock solution was prepared daily in dimethyl sulfoxide (DMSO) and was added to the pipette solution (200 *μ*g/mL) before the experiment and was replaced every 1–2 h. Extracellular and patch‐pipette solutions for single BK channel recordings contained (in mmol/L): 140 KCl, 1.08 MgCl_2_, 5 EGTA, 10 HEPES, and 3.16 CaCl_2_, adjusted to pH 7.2 with NaOH (Ca^2+^‐free concentration was calculated ~300 nmol/L with WEBMAXC Standard, http://www.stanford.edu/~cpatton/webmaxcS.htm, Chris Patton). 17*β*‐Estradiol and paxilline were applied by replacement of the extracellular solution via superfusion. 17*β*‐Estradiol and paxilline were purchased from Sigma‐Aldrich and were dissolved in DMSO. The final concentration of DMSO in the bath solution did not exceed 0.02%.

### Data analysis and statistics

Clampfit 10.3 (Molecular Device, Union City, CA) and Minianalysis software (Synaptosoft, Inc., NJ) were used to analyze the data. Mean values of the last 50 msec pulse of 200 msec depolarization step of at least five average files in the absence (control) and in the presence of 17*β*‐estradiol were analyzed to evaluate the effects of 17*β*‐estradiol on steady‐state whole‐cell BK currents. Only DSM cells with stable internal time controls of at least 8–10 min before application of 17*β*‐estradiol were used in this study. Five minutes of at least an 8–10 min stable patch‐clamp recording prior to application of 17*β*‐estradiol were analyzed for control data, and the last 5 min of continuous recordings of 10–15 min after application of 17*β*‐estradiol were analyzed to evaluate the effects of 17*β*‐estradiol on RMP, TBKCs, and single BK channel activity. The threshold of TBKCs was set at 9 pA. The values for single BK channel open probability (NPo) were obtained using the built‐in algorithm in Clampfit, which calculates as NP_O_ = (T_O_)/(T_O_+T_C_), where T_O_ and T_C_ correspond, respectively, to total open time and closed time during the recording interval. Single BK channel currents were not filtered with the software before data analysis. The single‐channel amplitudes were calculated from all‐point histograms using Gaussian distribution function to qualify the values for closed and open states.

Human DSM contractions were analyzed with MiniAnalysis software (Synaptosoft, Inc., NJ). The control values of each individual DSM strip were normalized and represented as 100%. GraphPad Prism 4.03 (GraphPad Software, Inc., La Jolla, CA) and CorelDRAW Graphics Suite X3 (Corel Co., Mountain View, CA) were used for the statistical analyses and data presentation. The data are expressed as mean±SEM for the “***n*** **”** (the number of DSM cells or strips) isolated from **“** ***N*** **”** (the number of patients). Statistical analyses were performed using the paired Student's *t* test or ANOVA with Bonferroni's post hoc test. A *P* value < 0.05 was considered statistically significant.

## Results

### 17β‐Estradiol increases depolarization‐induced steady‐state whole‐cell outward BK currents in freshly isolated human DSM cells

**Figure 1:**
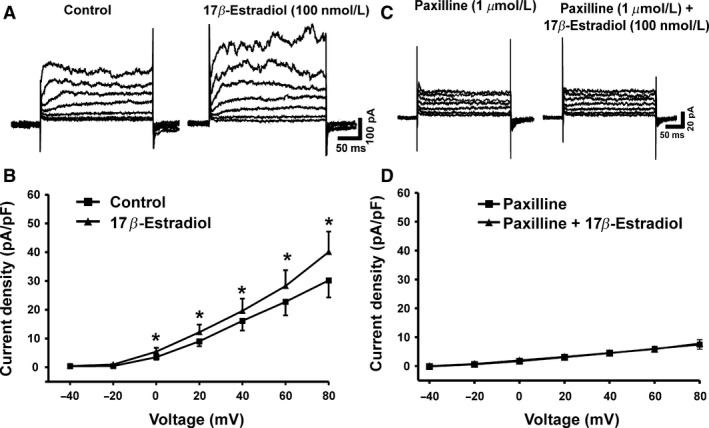
17*β*‐Estradiol increases the depolarization‐induced whole‐cell BK outward currents in freshly isolated detrusor smooth muscle (DSM) cells. (A) Representative original recordings illustrating the depolarization‐induced whole‐cell outward currents in the absence (control) and in the presence of 17*β*‐estradiol (100 nmol/L). (B) The current–voltage relationship curve summarizes the stimulatory effects of 17*β*‐estradiol on the whole‐cell outward currents (*n* = 12, *N* = 8; *P < 0.05). (C) Representative original recordings illustrating the depolarization‐induced whole‐cell outward currents in the presence of paxilline (1 *μ*mol/L) alone and in the presence of both paxilline (1 *μ*mol/L) and 17*β*‐estradiol (100 nmol/L). (D) The current–voltage relationship curve summarizes the lack of stimulatory effects of 17*β*‐estradiol on the whole‐cell outward currents in the presence of 1 *μ*mol/L paxilline (*n* = 6, *N* = 6; *P* > 0.05).

First, we investigated the regulatory role of 17*β*‐estradiol on human DSM cell excitability. We studied the effects of 17*β*‐estradiol on depolarization‐induced steady‐state whole‐cell outward BK currents by using amphotericin‐B‐perforated patch‐clamp and freshly isolated DSM cells. DSM cells used in this study had an average capacitance of 19.2 ± 1.2 pF (*n* = 43, *N* = 22). Whole‐cell outward K^+^ currents were increased gradually in response to voltage‐step depolarization from −40 mV to +80 mV as shown in Figure 1A. 17*β*‐Estradiol (100 nmol/L) significantly increased the whole‐cell K^+^ current density in DSM cells (Fig.**** 1). At the highest recording voltage of +80 mV, the whole‐cell outward K^+^ currents were 33.9 ± 6.5 pA/pF and 44.5 ± 7.8 pA/pF in the absence and in the presence of 17*β*‐estradiol, respectively (*n* = 12, *N* = 8; *P* < 0.05; Fig. 1A–B).

The effects of 17*β*‐estradiol on whole‐cell outward K^+^ currents were completely abolished by the selective BK channel inhibitor paxilline (1 *μ*mol/L). At +80 mV, the BK current amplitudes were 6.1 ± 1.7 pA/pF and 6.8 ± 1.8 pA/pF in the presence of 1 *μ*mol/L paxilline alone and in the presence of both paxilline (1 *μ*mol/L) and 17*β*‐estradiol (100 nmol/L), respectively (*n* = 6, *N* = 6; *P* > 0.05; Fig. 1C–D). The lack of 17*β*‐estradiol stimulatory effects on whole‐cell outward K^+^ currents in the presence of paxilline suggests that the potentiating effects of 17*β*‐estradiol involve exclusively BK channel activation.

### 17*β*‐Estradiol increases TBKC activity in freshly isolated DSM cells

**Figure 2:**
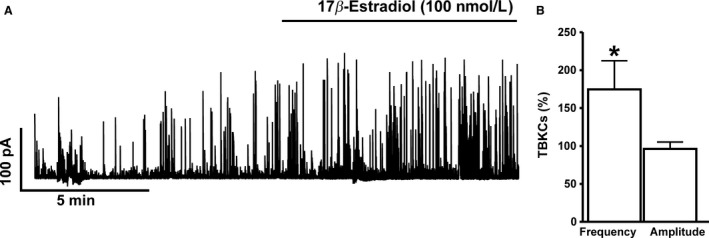
17*β*‐Estradiol increases transient BK current (TBKC) activity in freshly isolated detrusor smooth muscle (DSM) cells. (A) A representative original recording illustrating the stimulatory effect of 100 nmol/L 17*β*‐estradiol on the frequency of TBKCs in human DSM single cell. (B) Summary data illustrating the stimulatory effects of 17*β*‐estradiol (100 nmol/L) on the frequency of TBKCs (*n* = 8, *N* = 5; *P < 0.05). The data were normalized to control values (prior to 17*β*‐estradiol addition) taken as 100% and were presented as percentages (%). TBKCs were recorded at a holding potential of −20 mV.

DSM cells generate TBKCs, caused by localized Ca^2+^ release events from the sarcoplasmic reticulum, known as Ca^2+^ sparks, which leads to subsequent BK channel activation (Petkov 2014). At a holding potential of −20 mV, 17*β*‐estradiol (100 nmol/L) increased the frequency of TBKCs by 74.6 ± 37.8% without significantly altering the average TBKC amplitude (*n* = 8, *N* = 5; *P* < 0.05; Fig. 2). The results suggest that activation of BK channels with 17*β*‐estradiol enhances TBKC frequency in DSM cells.

### 17*β*‐Estradiol increases single BK channel open probability in the excised membrane patches of human DSM cells

**Figure 3:**
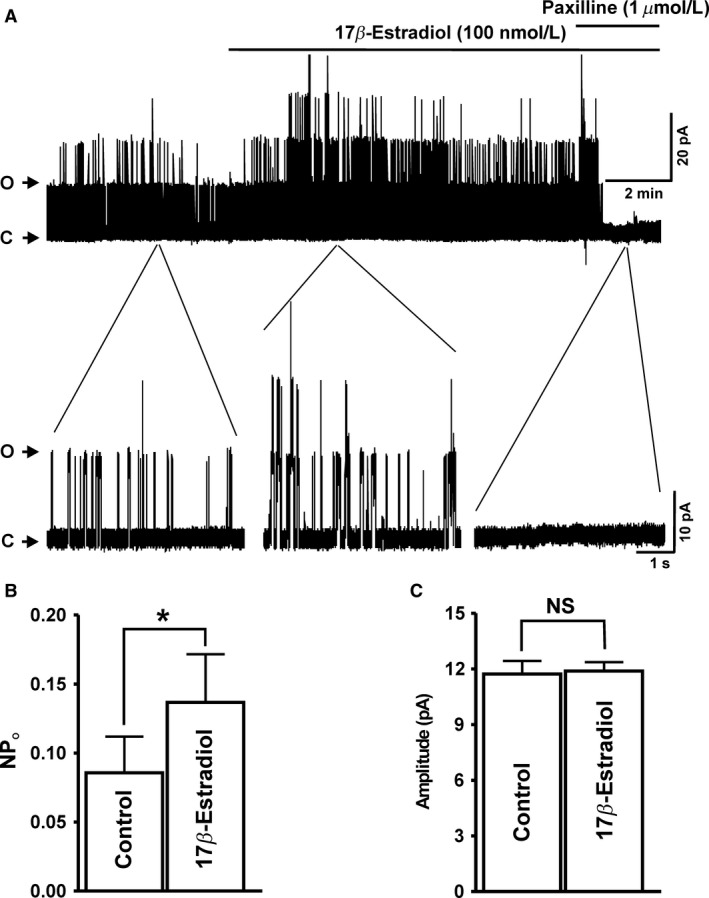
17*β*‐Estradiol increases the single BK channel open probability (NPo) of human detrusor smooth muscle (DSM) excised membrane patches. (A) A representative original recording from excised membrane patch of a DSM cell illustrating the stimulatory effect of 17*β*‐estradiol (100 nmol/L) on NPo in inside‐out configuration. A portion of recording before and after application of 17*β*‐estradiol (100 nmol/L) is shown in expanded time scales. (B and C) Summary data illustrating the stimulatory effects of 17*β*‐estradiol (100 nmol/L) on NPo (B) and single BK channel current amplitude (C) observed in inside‐out patches (*n* = 9, *N* = 7; *P < 0.05). Posttreatment of DSM cell membrane patches with paxilline (1 *μ*mol/L) completely abolishes the NPo of single BK channels (*n* = 5, *N* = 5; *P < 0.05). ‘C’ and ‘O’ represent the closed and opened states of BK channels, respectively.

To further investigate the precise cellular mechanism by which 17*β*‐estradiol activates BK channels in DSM cells, we conducted a series of experiments on single BK channel activity by using the inside‐out excised‐patch configuration of the patch‐clamp technique. 17*β*‐Estradiol (100 nmol/L) significantly increased the single BK channel NPo from 0.086 ± 0.030 to 0.137 ± 0.030 (*n* = 9, *N* = 7, *P* < 0.05; Fig.**** 3). However, 17*β*‐estradiol (100 nmol/L) did not affect the single BK channel current amplitude, which was 11.7 ± 0.7 pA and 11.9 ± 0.5 pA in the absence and presence of 17*β*‐estradiol, respectively (*n* = 9, *N* = 7; *P* > 0.05; Fig. 3). As illustrated in Figure 3A, the selective BK channel inhibitor paxilline (1 *μ*mol/L) completely abolished single BK channel activity (*n* = 5, *N* = 5; *P* < 0.05), suggesting that the stimulatory effect of 17*β*‐estradiol on channel NPo was due to BK channel activation. The results from these experiments support the concept that 17*β*‐estradiol rapidly activates the BK channels by directly targeting the channel, rather than involving intracellular signaling pathways.

### BK channel activation with 17*β*‐estradiol hyperpolarizes the resting membrane potential (RMP) in DSM cells

**Figure 4:**
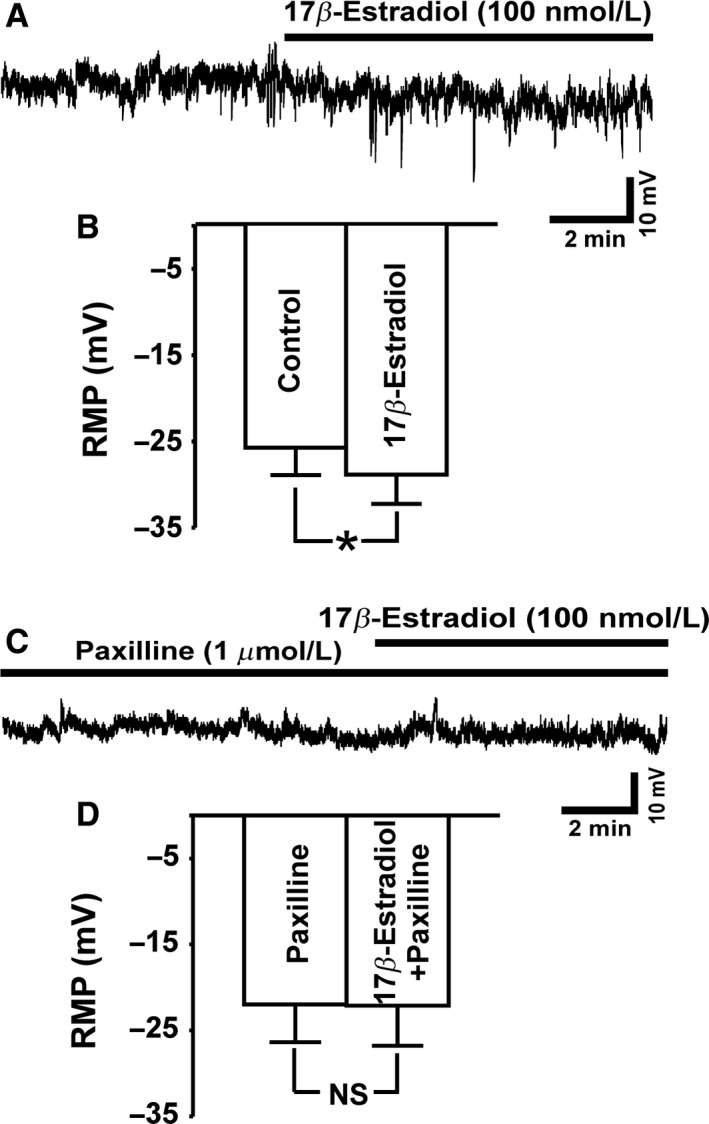
17*β*‐Estradiol hyperpolarizes the resting membrane potential (RMP) in detrusor smooth muscle (DSM) cells. (A) A representative trace of an RMP recording in current‐clamp mode illustrating the hyperpolarizing effects of 17*β*‐estradiol (100 nmol/L) in an isolated human DSM cell. (B) Summary data illustrating the hyperpolarizing effects of 17*β*‐estradiol on the human DSM cell RMP (*n* = 11, *N* = 9; *P < 0.05). (C) A representative trace of an RMP in current‐clamp mode demonstrating that when the BK channels were blocked with 1 *μ*mol/L paxilline, 17*β*‐estradiol (100 nmol/L) did not cause membrane hyperpolarization in DSM cells. (D) Summary data illustrating that 17*β*‐estradiol had no effect on the human DSM cell RMP in the presence of 1 *μ*mol/L paxilline (*n* = 8, *N* = 5; *P* > 0.05).

Next, we aimed to investigate the BK channel‐dependent regulation of the human DSM cell RMP by 17*β*‐estradiol. The current‐clamp experiments showed that 17*β*‐estradiol (100 nmol/L) significantly hyperpolarized the DSM cell RMP by ~3 mV, from a control value of −25.7 ± 3.2 mV to −28.9 ± 3.3 mV in the presence of 17*β*‐estradiol (*n* = 11, *N* = 9; *P* < 0.05; Fig. 4A–B). As shown in Figure 4C**, 17** *β*‐estradiol had no effect on the human DSM cell RMP when administered in the presence of the BK channel inhibitor paxilline (1 *μ*mol/L); with the RMP of **−**21.9 ± 4.4 mV, which was practically identical with the control value (paxilline only) of **−**22.1 ± 4.7 mV (*n* = 8, *N* = 5; *P* > 0.05; Fig. 4D). These data support the concept that 17*β*‐estradiol regulates the human DSM cell RMP through a BK channel‐dependent mechanism.

### 17β‐Estradiol reduces spontaneous phasic contractions of human DSM‐isolated strips in a BK channel‐dependent manner

**Figure 5:**
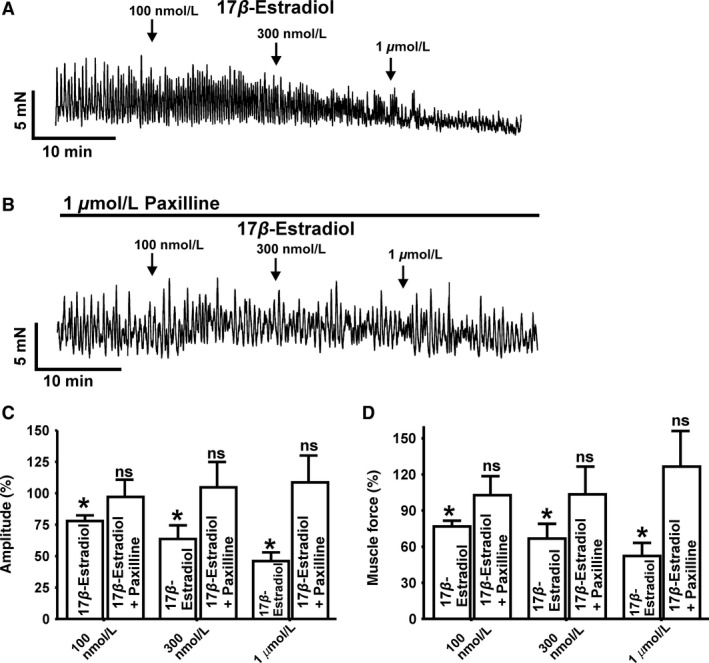
17*β*‐Estradiol inhibits spontaneous phasic contractions of human detrusor smooth muscle (DSM)‐isolated strips. (A) Representative original recordings illustrating the concentration‐dependent inhibitory effects of 17*β*‐estradiol (100 nmol/L‐1 *μ*mol/L) on spontaneous phasic contractions of isolated DSM strips in the absence (A) or presence (B) of paxilline (1 *μ*mol/L). (C and D) Summary data illustrating the inhibitory effects of 17*β*‐estradiol on the amplitudes and muscle force of spontaneous phasic contraction of DSM‐isolated strips in the absence (*n* = 8, *N* = 5) and presence of 1 *μ*mol/L paxilline (*n* = 5, *N* = 5; *P < 0.05).

To investigate the regulatory role of 17*β*‐estradiol on human DSM contractility, we performed functional studies with human DSM‐isolated strips. 17*β*‐Estradiol (100 nmol/L‐1 *μ*mol/L) significantly inhibited the spontaneous phasic contraction amplitude and muscle force integral (defined as area under the time–force curve) in a concentration‐dependent manner (*n* = 8, *N* = 5; *P* < 0.05; Fig. 5). 17*β*‐Estradiol (1 *μ*mol/L) decreased human DSM spontaneous phasic contraction amplitude and muscle force integral by 53.3 ± 8.9% and 44.4 ± 13.2%, respectively (*n* = 8, *N* = 5; *P* < 0.05; Fig. 5), without significantly changing phasic contraction frequency (*n* = 8, *N* = 5; *P* > 0.05). The inhibitory effect of 17*β*‐estradiol on human DSM contractility was abolished by 1 *μ*mol/L paxilline, a selective BK channel inhibitor. As shown in Figure 5 **,** in DSM strips preincubated with the selective BK channel inhibitor paxilline (1 *μ*mol/L), 17*β*‐estradiol (100 nmol/L–1 *μ*mol/L) did not affect spontaneous phasic contraction amplitude and muscle force integral (*n* = 5, *N* = 5; *P* > 0.05, Fig. 5B–D). The data suggest that 17*β*‐estradiol decreases spontaneous phasic contractions in human DSM, and this effect is mediated by BK channels.

### 17β‐Estradiol reduces nerve‐evoked contractions of human DSM‐isolated strips

**Figure 6:**
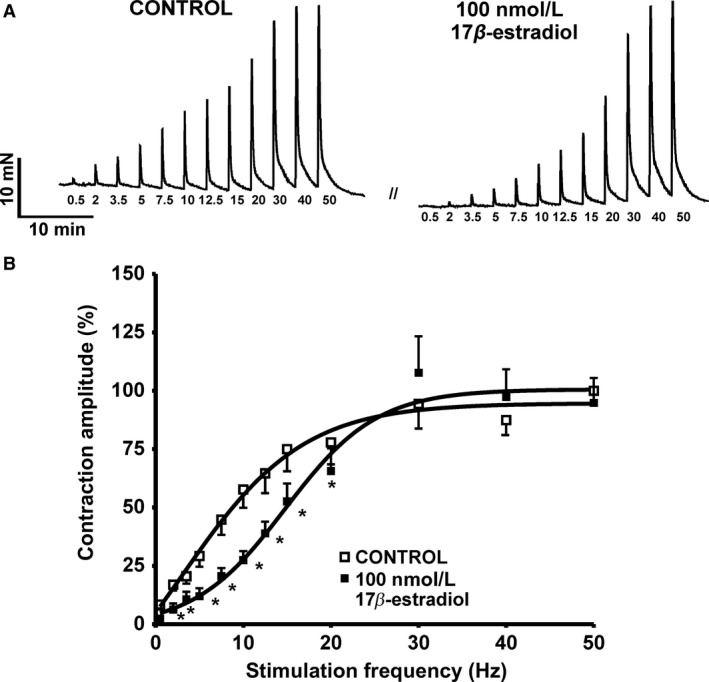
17*β*‐Estradiol inhibits the nerve‐evoked contractions of human detrusor smooth muscle (DSM)‐isolated strips. (A) Representative original traces illustrating the inhibitory effect of 17*β*‐estradiol (100 nmol/L) on the EFS‐induced contraction of DSM‐isolated strips. (B) Summary data illustrating the inhibitory effects of 17*β*‐estradiol on the amplitude of the EFS‐induced contractions of DSM‐isolated strips (*n* = 7, *N* = 3; *P < 0.05). The data were normalized to contraction amplitude at the stimulation frequency of 50 Hz (prior to 17*β*‐estradiol addition) taken as 100% and were presented as percentages (%).

In the next series of isometric DSM tension recording experiments, we tested the effects of 17*β*‐estradiol on the nerve‐evoked (EFS‐induced) contractions induced by electrical field stimulation (EFS) at frequencies ranging 0.5–50.0 Hz. The EFS pulse parameters were as follows: 0.75‐msec pulse width, 20‐V pulse amplitude, and 3‐sec stimulus duration**.** As shown in Figure**** 6, 17*β*‐estradiol (100 nmol/L) significantly inhibited the EFS‐induced contractions, in particular, at lower stimulation frequencies (0.5–20.0 Hz) (*n* = 7, *N* = 3; *P* < 0.05).

## Discussion

BK channels are key determinants of human DSM function and their activity is dually controlled by intracellular Ca^2+^ and voltage (Hristov et al. 2011; Petkov 2012, 2014). Until now, the regulatory role of estrogens on BK channel activity has never been investigated in human DSM. Detailed knowledge about the regulation of DSM excitability and contractility by estrogens is particularly important in human DSM, as humans are the target species for therapeutic interventions. Substantial interspecies differences in the anatomy and physiology of the lower urinary tract are well documented. Therefore, the information obtained from experimental animal models cannot be directly translated to humans. Here, we investigated the underlying molecular and cellular mechanism by which 17*β*‐estradiol regulates human DSM function. We utilized a combined experimental approach using nanomolar concentrations (100 nmol/L) of 17*β*‐estradiol to examine its physiological role in regulating excitation–contraction coupling in human DSM. A key aspect of this study is that for the first time, the effects of 17*β*‐estradiol on BK channel activation have been elucidated directly in clinically characterized human DSM at both the cellular and tissue levels. We provided compelling novel evidence to support the concept that under physiological conditions, 17*β*‐estradiol decreases human DSM excitability and contractility through a mechanism involving direct BK channel activation.

In non‐DSM smooth muscle, such as bovine aortic (Valverde et al. 1999), canine colonic (Dick et al. 2001), and human coronary artery (White et al. 2002), 17*β*‐estradiol increased BK channel activity through a direct interaction with the regulatory *β*_1_‐subunit of the BK channel. In DSM, this mechanism could have an important physiological role, as the BK channel *β*_1_‐subunit is critical for DSM contractility (Petkov et al. 2001). A recent study in guinea pig DSM is in support of this hypothesis, and further revealed that 17*β*‐estradiol activates whole‐cell BK currents, TBKCs, and hyperpolarizes the DSM cell RMP via direct BK channel stimulation (Provence et al. 2015). Consistent with these findings, our data from whole‐cell patch‐clamp recordings showed that 17*β*‐estradiol increased depolarization‐induced steady‐state whole‐cell K^+^ currents in freshly isolated human DSM cells. These stimulatory effects of 17*β*‐estradiol were abolished by the selective BK channel inhibitor paxilline, suggesting a mediatory role of the BK channels (Fig. 1).

It is important to consider that the underlying cellular mechanism of 17*β*‐estradiol‐induced BK channel activation may include direct stimulation of the BK channel, or indirect pathways including modulation of intracellular Ca^2+^ dynamics that affect BK channel Ca^2+^ sensitivity. Single BK channel recordings from inside‐out excised membrane patches under the conditions of constant [Ca^2+^] exposure of ~ 300 nmol/L from guinea pig DSM cells have shown 17*β*‐estradiol to significantly increased BK channel NPo, confirming a direct nongenomic mechanism of 17*β*‐estradiol in the regulation of BK channel activity (Provence et al. 2015). In line with this recent study, our data from human DSM inside‐out excised membrane patches revealed a significant increase in BK channel NPo by 17*β*‐estradiol (Fig. 3A–B), without affecting the single BK channel amplitude (Fig.**** 3 **C**). Two important conclusions can be drawn from these observations. First, the rapid stimulatory effects of 17*β*‐estradiol on human DSM BK channel activity indicate that 17*β*‐estradiol regulates BK channel activity through a mechanism independent of the genomic estrogen receptors. Second, the stimulatory effects of 17*β*‐estradiol on BK channel NPo (Fig.**** 3A–B) are consistent with direct modulation of the smooth muscle‐specific regulatory BK channel *β*_1_‐subunit by 17*β*‐estradiol. The molecular mechanism by which 17*β*‐estradiol elevates NPo may be attributed to either increase in BK channel open probability or increase in BK channel number. Regardless of the precise molecular mechanism by which 17*β*‐estradiol increase NPo, our single‐channel experiments clearly demonstrate BK channel activation in the absence of intracellular signaling pathways.

The single‐channel experiments (Fig.**** 3) could explain why 17*β*‐estradiol increases the frequency of TBKCs in DSM cells, without significant effects on TBKC amplitude (Fig. 2). The increase in TBKC frequency could be attributed to an increase in BK channel NPo by 17*β*‐estradiol (Fig. 3), which can thus increase the number of single BK channel opening events at −20 mV in DSM cells. Therefore, 17*β*‐estradiol increased the number of small BK channel opening events reaching the TBKC threshold of 9 pA, (see Materials and Methods) as a result of direct modulation of BK channel NPo, while having no potentiating effects on the average amplitude of TBKCs in DSM cells.

The BK channel *β*_1_‐subunit, known to be highly expressed in human DSM (Hristov et al. 2011), is involved in regulation of DSM contractility (Petkov et al. 2001). Therefore, modulation of BK channel activity via the *β*_1_‐subunit may have clinical relevance in the treatment of OAB. 17*β*‐Estradiol can modulate BK channel activity by binding to the regulatory BK channel *β*_1_‐subunit (Valverde et al. 1999; De Wet et al. 2006) causing direct channel activation. Here, for the first time we provided evidence that 17*β*‐estradiol inhibits human DSM cell excitability and contractility by direct BK channel activation. This cellular mechanism should be considered when novel hormonal therapies for bladder dysfunction are being developed.

On the other hand, our study cannot completely exclude alternative contributing mechanisms including 17*β*‐estradiol‐induced perturbation of the membrane environment surrounding the BK channel. Many different types of ion channels, including the BK channel, are known to be modulated by fatty acids, lipids, and other membrane active agents. In addition, estrogens can also potentially affect BK channel expression and splice variants indirectly by genomic mechanisms (Kundu et al. 2007). Whether these alternative mechanisms operate in parallel to precisely control excitability and contractility in human DSM remains to be investigated.

It is well known that the membrane potential of human DSM cell is actively regulated by the BK channels (Hristov et al. 2011, 2012). Inhibition of the BK channels leads to DSM cell membrane depolarization (Hristov et al. 2011), while pharmacological activation of the BK channel with selective channel openers results in DSM cell membrane hyperpolarization (Hristov et al. 2012). Similarly, in rodent DSM cells 17*β*‐estradiol hyperpolarizes DSM cell membrane (Provence et al. 2015). Here, for the first time in human DSM cells, we demonstrated that activation of BK channels with 17*β*‐estradiol significantly hyperpolarized the RMP in a BK‐channel‐dependent manner (Fig.**** 4). These results are consistent with previous findings in guinea pig DSM (Provence et al. 2015), and clearly demonstrate a regulatory role for 17*β*‐estradiol on the human DSM cell RMP.

DSM cell membrane hyperpolarization attenuates L‐type voltage‐gated Ca^2+^ channel activity, decreases intracellular Ca^2+^ concentrations, thus causing DSM relaxation (Hristov et al. 2012). Our data suggest that under physiological condition estrogens are essential modulators of the human DSM RMP (Fig.**** 4), which in turn affects DSM contractility. Indeed, our functional studies of DSM contractility showed that 17*β*‐estradiol significantly inhibited the amplitude and force of spontaneous phasic contractions (Fig. 5) in human DSM‐isolated strips. Paxilline, a selective BK channel blocker, prevented the relaxant effect of 17*β*‐estradiol on spontaneous phasic contractility suggesting that 17*β*‐estradiol inhibits human DSM contractions primarily by its stimulatory effects on BK channel activity (Fig. 5).

We have designed the EFS experiments to assess the effects of 17*β*‐estradiol on nerve‐evoked contractions over a large range of stimulation frequencies (0.5–50.0 Hz). Our results showed that in human DSM, 17*β*‐estradiol reduced EFS‐induced contractions, particularly at the lower and more physiologically relevant stimulation frequencies **(**Fig. 6 **)**, indicating that 17*β*‐estradiol regulates nerve‐evoked human DSM contractions. Therefore, targeting the BK channels with 17*β*‐estradiol could represent a novel pharmacological approach to control neurogenically mediated detrusor dysfunction in female patients.

It has been shown that high, nonphysiological concentrations of 17*β*‐estradiol (30 *μ*mol/L) cause a decrease in the pharmacologically induced and nerve‐evoked contractions in rat DSM‐isolated strips (Valeri et al. 2009). In another study, 17*β*‐estradiol (30 *μ*mol/L) induced inhibitory effects on the KCl‐ and muscarinic receptor‐induced contractions of pig DSM‐isolated strips (Dambros et al. 2004). Estrogen receptor antagonists did not affect the 17*β*‐estradiol‐induced inhibitory effects on the pharmacologically induced DSM contractions suggesting no functional role for the estrogen receptors in mediating 17*β*‐estradiol inhibitory effects on DSM contractions (Dambros et al. 2004).

In this study, we have demonstrated that 17*β*‐estradiol decreases both spontaneous and nerve‐evoked human DSM contractions at nanomolar concentrations (Figs.**** 5 and 6). These concentrations are significantly below that of earlier reports that used nonphysiological micromolar concentrations of 17*β*‐estradiol (Dambros et al. 2004; Valeri et al. 2009). Therefore, our data support the concept that 17*β*‐estradiol may regulate human DSM contractility under physiological conditions.

In conclusion, for the first time directly in human DSM, our results reveal that 17*β*‐estradiol regulates DSM excitability and contractility in a BK‐channel‐dependent manner. The study provides compelling evidence that 17*β*‐estradiol exhibited direct nongenomic stimulatory effects on BK channels, thus decreasing the excitability and contractility of human DSM. The combined results support the idea that activation of BK channels with estrogens may represent a novel and effective treatment for patients with OAB and associated detrusor overactivity.

## Conflict of Interest

The authors declare no conflicts of interest.
